# Targeting IspD for Anti-infective and Herbicide Development: Exploring Its Role, Mechanism, and Structural Insights

**DOI:** 10.1021/acs.jmedchem.4c01146

**Published:** 2025-01-03

**Authors:** Daan Willocx, Eleonora Diamanti, Anna K. H. Hirsch

**Affiliations:** † Helmholtz Institute for Pharmaceutical Research (HIPS)−Helmholtz Centre for Infection Research (HZI), Saar-land University, Campus E8.1, 66123Saarbrücken, Germany; ‡ Helmholtz International Lab for Anti-Infectives, Saarland University, Campus E8.1, 66123Saarbrücken, Germany; ¶ Department of Pharmacy, Saarland University, Campus E8.1, 66123Saarbrücken, Germany

## Abstract

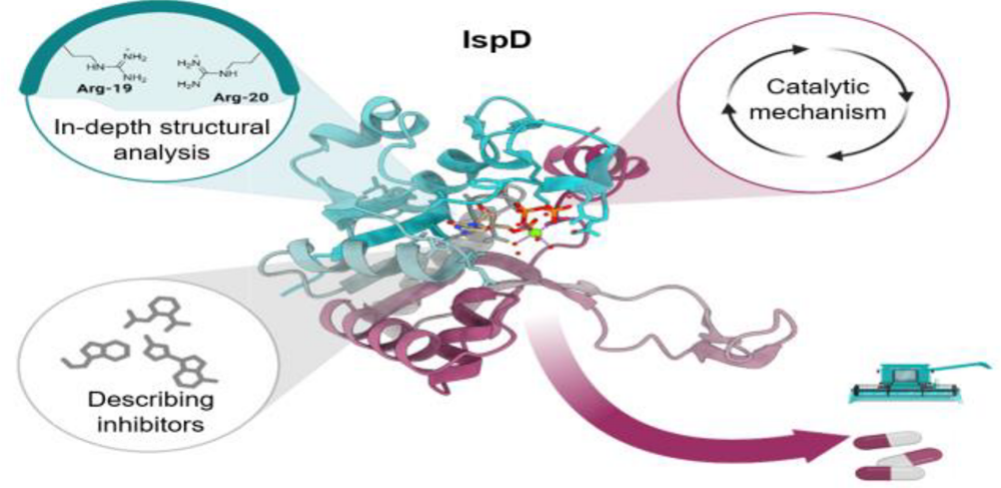

Antimicrobial resistance (AMR) and herbicide resistance pose threats to society, necessitating novel anti-infectives and herbicides exploiting untapped modes of action like inhibition of IspD, the third enzyme in the MEP pathway. The MEP pathway is essential for a wide variety of human pathogens, including *Pseudomonas aeruginosa*, *Mycobacterium tuberculosis*, and *Plasmodium falciparum,* as well as plants. Within the current perspective, we focused our attention on the third enzyme in this pathway, IspD, offering a comprehensive summary of the reported modes of inhibition and common trends, with the goal to inspire future research dedicated to this underexplored target. In addition, we included an overview of the history, catalytic mechanism, and structure of the enzyme to facilitate access to this attractive target.

## Significance

Herbicide and anti-infective resistance is an increasing global concern. The enzymes of the methyl-D-erythritol phosphate (MEP) pathway, including IspD, offer promising targets for the development of novel anti-infectives and herbicides. This Perspective delves into the history, catalytic mechanism, structural intricacies, and inhibitors of IspD. Our aim is to introduce new researchers to IspD and to inspire further exploration by experienced scientists.

## Introduction

**Scheme 1:**
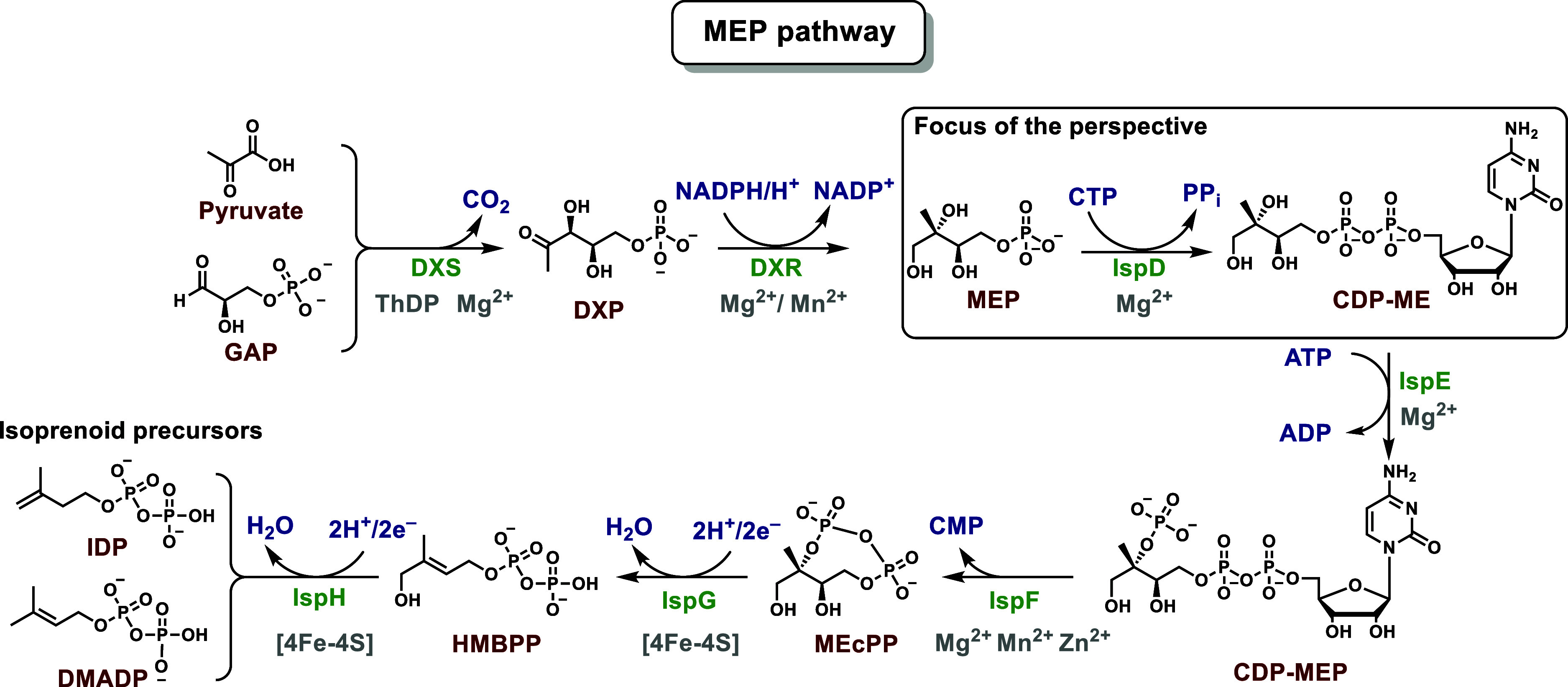
Overview of the MEP Pathway

Both herbicides and anti-infectives are indispensable pillars of modern civilization based on their revolutionary impact. While anti-infectives allow safe and effective treatment of infectious diseases, herbicides make harvests more reliable and enhance crop yields.^1,2^ Despite their accomplishments, both are prone to resistance development, which has become increasingly problematic in recent years. For example, in 2019 alone, an estimated 4.95 million deaths were attributed to antimicrobial resistance.^3^ Hence, new anti-infectives and herbicides with new modes of action (MOAs) are urgently needed.^4^ In this regard, the 2-*C*-methylerythritol-d-erythritol-4-phosphate (MEP) pathway is a rich source of attractive targets (Scheme 1). The pathway ensures the biosynthesis of the essential isoprenoid precursors isopentenyl diphosphate (IDP) and dimethylallyl diphosphate (DMADP) and is constituted by seven enzymes (Scheme 1).^5^ Previous literature reports demonstrate the dependency of plants and many human pathogens on the MEP pathway, including *Mycobacterium tuberculosis* (*Mt*), *Plasmodium* parasites, and Gram-negative bacteria such as *Pseudomonas aeruginosa* (*Pa*) (Tables 1, 2).^6−10^ Importantly, the pathway is absent in human cells, reducing the risk of off-target-based side effects.^11−14^ We have previously analyzed the druggability of the MEP pathway enzymes *in silico* by utilizing DoGSiteScorer, a web-based tool designed to identify and characterize potential binding pockets and subpockets.^11,15^ The evaluation supports the potential druggability of all substrate- and cofactor-binding pockets of the enzymes. Targeting these active sites is, however, challenging, as most are highly polar as a consequence of the hydrophilic phosphorylated intermediates. Nonetheless, most enzymes feature allosteric pockets with more favorable hydrophobic character. For example, targeting the allosteric pocket in *Arabidopsis thaliana* IspD (*At*IspD) led to a new class of inhibitors with nanomolar activity.^14^ In this perspective, we will focus on the third enzyme in the MEP pathway, namely, 4-diphosphocytidyl-2*C*-methyl-d-erythritol synthase (IspD) (Scheme 1). The enzyme is encoded on the *ygbp* gene and catalyzes the conversion of MEP and CTP toward 4-diphosphocytidyl-2*C*-methyl-d-erythritol (CDP-ME) and diphosphate.^16^ Regardless of the widespread occurrence of the MEP pathway (Table 1), only a handful of IspD homologues were confirmed to be inhibited by small-molecule inhibitors, again highlighting the underexplored nature of this attractive target. In the following, the discovery, mechanism, and structure of IspD are discussed as well as all known inhibitors and their binding mode. We aim to provide a deeper understanding of the target in general and inspiration for the design of future generations of inhibitors.

**Table 1:** Prevalence of the MEP Pathway^78^

Gram-positive	Gram-negative
*Staphylooccus aureus*	*Chlamydia pneumoniae*
*Bacillus anthracis*	*Pseudomonas aeruginosa*
*Clostridium difficile*	*Klebsiella pneumoniae*
*Listeria monocytogenes*	*Haemophilus influenzae*
*Bacillus subtilis*	*Vibrio cholerae*

**Table 2:** Examples of IspD Homologues and the Potential Application of Their Inhibitor

Abbreviation	Origin	Potential inhibitor application
*At*IspD	*Arabidopsis thaliana*	Herbicide
*Bs*IspD	*Bacillus subtilis*	Model organism
*Ec*IspD	*Escherichia coli*	Anti-infective
*Hs*IspD	*Human*	Understanding the Walker–Warburg syndrome
*Ms*IspD	*Mycobacterium smegmatis*	Research toward the *Mycobacterium* genus
*Mt*IspD	*Mycobacterium tuberculosis*	Antitubercular drugs
*Pf*IspD	*Plasmodium falciparum*	Antimalarial
*Pv*IspD	*Plasmodium vivax*	Antimalarial

## Discovery of the MEP Pathway

**Figure 1:**
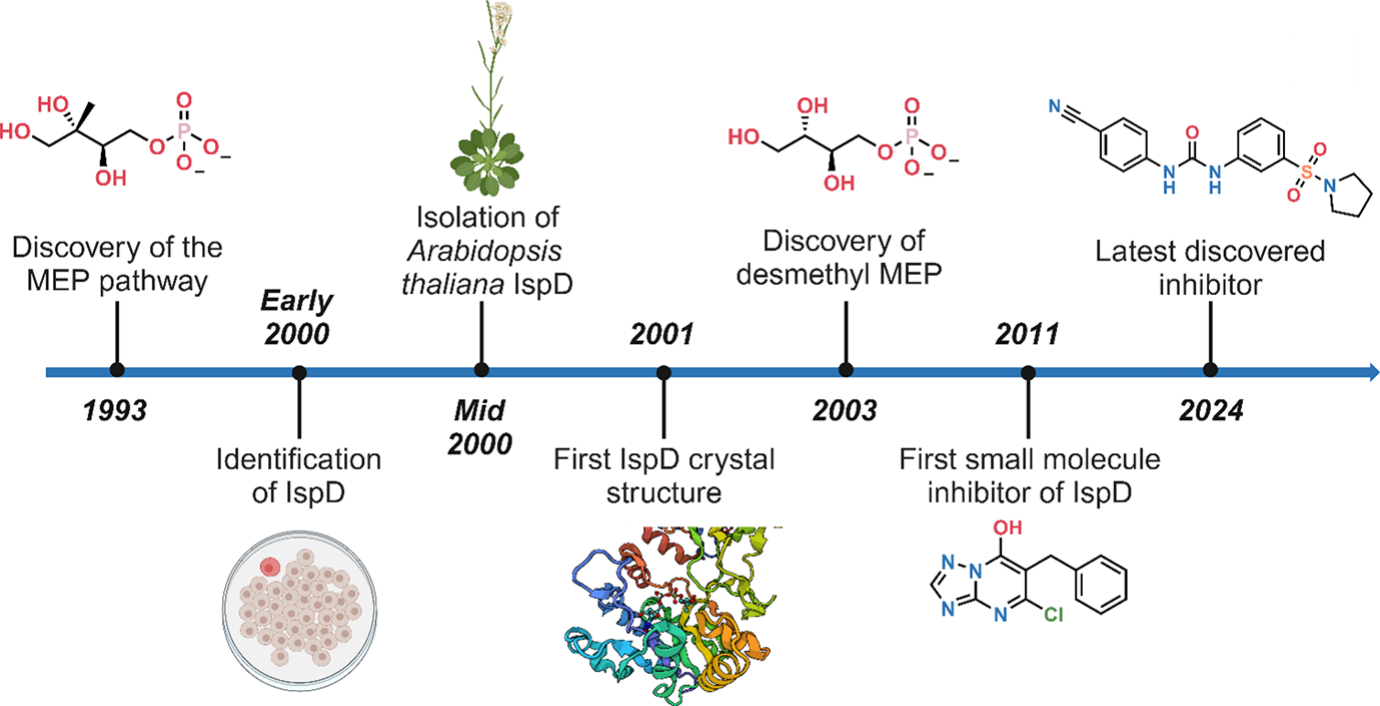
IspD research from initial discovery to different homologues, mechanism, and inhibitors. methyl-D-erythritol phosphate (MEP)^14,54,79^

The isoprenoids are one of the largest classes of natural products comprising over 50,000 structurally diverse members.^13^ All of them share IDP and DMADP as common precursors, which are linked together via condensation reactions. Functionalization of the linked precursors with moieties such as alcohols, aldehydes, and esters, leads to great diversity within this class of biomolecules.^17,18^ Until the mid-1990s, there was a consensus among scientists that the mevalonate pathway (MVA) was the only biosynthetic pathway for organisms to produce DMADP and IDP. Conflicting results in ^13^C-labeling experiments concerning isoprenoid biosynthesis, however, hinted at the existence of an additional pathway. The MEP pathway was later independently discovered by the research groups of Rohmer and Arigoni.^13,19−22^ By using ^13^C-labeled glucose in isotopic feeding experiments, two alternative starting molecules could be identified, namely, glyceraldehyde-3-phosphate (GAP) and pyruvate.^21^ The assignment of pyruvate and GAP as precursors led to the identification of the first intermediate in the new pathway, the unbranched 1-deoxyxylulose 5-phosphate (DXP). Only three years after the initial discovery, the third enzyme in this cascade was uncovered by Kuzuyama *et al*.^23^ At that point in time, only the first two enzymes, DXS and DXR, and their substrates DXP and MEP were known. The other enzymes were discovered by the same group using *Escherichia coli* (*Ec*) transformants. Their experiments led to the identification of an enzyme, IspD, with the ability of transforming MEP in the presence of CTP, toward an unknown product. After characterization, the product could be identified as CDP-ME.^23^ Shortly after the discovery of *Ec*IspD, Rohdich F. *et al.* isolated the first plant-based IspD from *A. thaliana* (Figure 1).^24^ In the following years, the whole pathway was characterized, leading to the discovery of a total of seven enzymes that catalyze what is now known as the MEP pathway.

### Target Validation of the Enzymes of the MEP Pathway

The MEP pathway enzymes have been thoroughly studied in the years after their discovery. It became clear that disruptions in the MEP pathway enzymes resulted in lethality for various organisms, including *E. coli* and *M. tuberculosis*.^6,25,26^ The MEP pathway and its products were also found to be essential during the entire life cycle of the *Plasmodium* parasites. Interestingly, within these parasites, the pathway takes place in the apicoplast, a nonphotosynthetic plastid-like organelle of prokaryotic origin, instead of the cytosol where most other processes occur.^27−31^ Plants, however, utilize the MVA and MEP pathways simultaneously, and research has shown that each pathway is essential for their survival. They take place in different compartments, with the MVA pathway localized in the cytosol and the MEP pathway in the plastids. Even though both pathways afford identical isoprenoid precursors, these, in turn, lead to structurally divers isoprenoids. For example, the MEP pathway isoprenoid precursors are used for the production of chlorophyll and carotenoids, while most sterols originate from MVA-derived precursors.^17^ The combination of all these discoveries highlight the unique features making the consituent enzymes of the MEP pathway attractive targets for drug and herbicide development.

### The Catalytic Mechanism of IspD

**Scheme 2:**
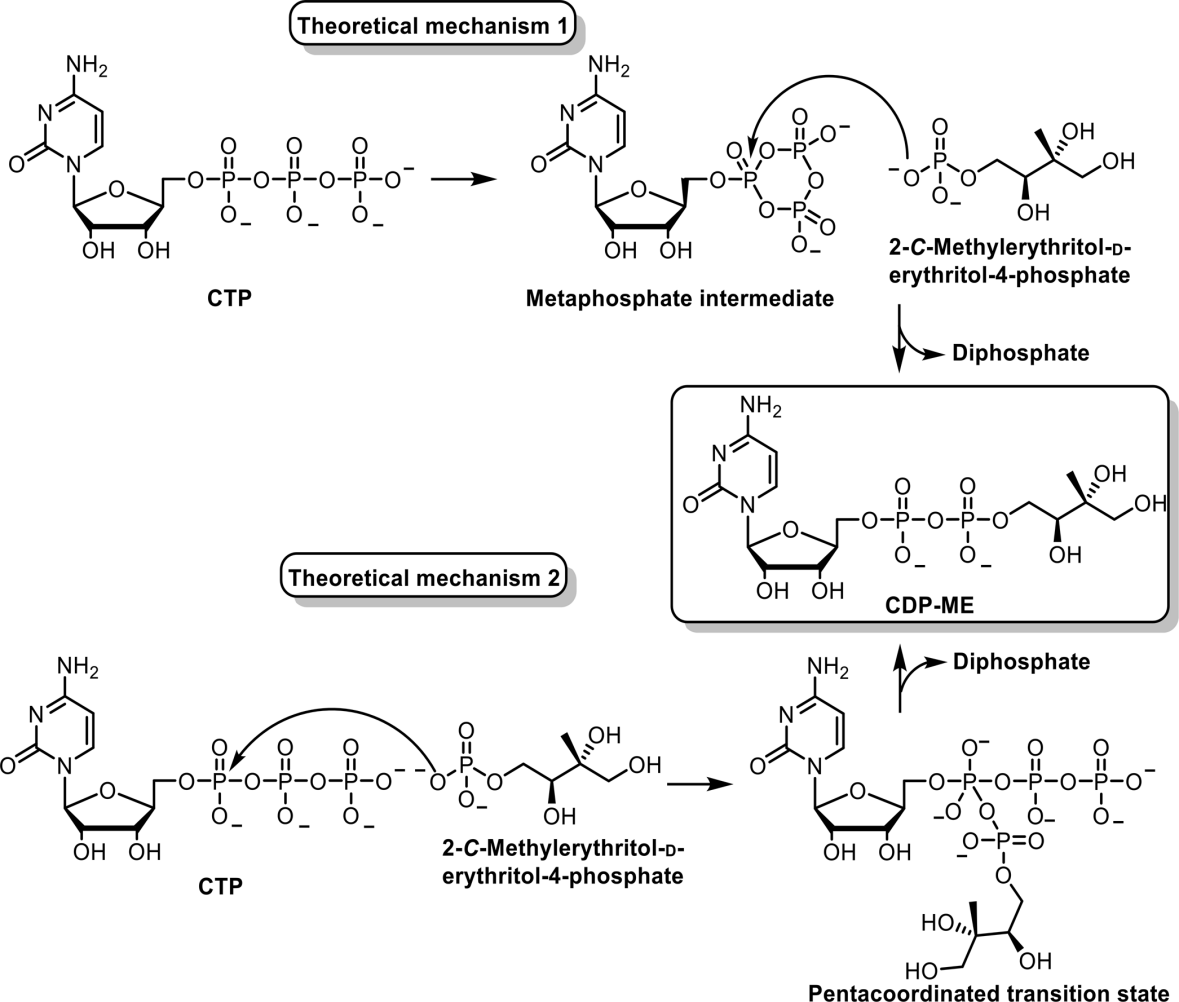
Representation of the Proposed Reaction Mechanisms Catalyzed by IspD, Leading to the Formation of 4-Diphosphocytidyl-2*C*-methyl-d-erythritol (CDP-ME)

Two theoretical mechanisms for cytidyl transferases have been proposed. The first starts with the formation of a highly reactive metaphosphate intermediate at the α-phosphate from CTP. Next, the intermediate undergoes a nucleophilic attack from the 4-phosphate from MEP directed at the α-phosphate of CTP affording CDP-ME and releasing diphosphate (Scheme 2, top). The second mechanism starts with the nucleophilic attack of the 4-phosphate of MEP onto the α-phosphate of CTP, resulting in an unstable pentacoordinated negatively charged transition state, which subsequently collapses in CDP-ME with the release of diphosphate (Scheme 2, bottom). Experimental data have yet to be acquired to unambiguously confirm the correct mechanism. Nevertheless, current crystallographic data on *apo*-IspD and the IspD–CTP complex suggest that the protein surface rearranges upon CTP binding, meaning that there are plenty of positively charged amino acid side chains stabilizing the pentavalent transition state in addition to the cation. Furthermore, the α-phosphate of CTP is displaced to be in the proximity of MEP to undergo nucleophilic attack, favoring the second reaction mechanism.^32^ Further studies to unravel the sequence of the mechanism were performed by pulse-chase experiments by Richard *et al*.^33^ Their observations point toward a sequential mechanism in which CTP must bind to the enzyme before MEP. Observations pointing in the same direction were found by Seemann and co-workers after performing bisubstrate kinetic analysis, finding the dissociation constant of MEP from the IspD-CTP complex to be 20 μM, which is 13 times lower than the dissociation constant for the MEP–IspD complex (265 μM).^34^ Further investigations toward the catalytic mechanism uncovered a clear preference for CTP as a nucleotide 5-triphosphate.^35^ Other nucleotide 5-triphosphates such as adenosine triphosphate, guanosine triphosphate, and uridine triphosphate exhibited either significantly reduced turnover or no turnover at all. The selectivity is attributed to the compact pocket in which the nucleotide base resides during catalytic action. As a cofactor, the divalent Mg^2+^ cation yields the highest activity, although the use of Mn^2+^ and Co^2+^ also led to formation of CDP-ME. Conversely, the use of other divalent cations, such as Cu^2+^, Ni^2+^, Ca^2+^, Fe^2+^, or Zn^2+^, rendered IspD inactive. Michaelis constants (*K*_m_) for both MEP and CTP of several IspD homologues demonstrate similar values for all homologues, with the only inconsistency being the *K*_m_ value for MEP of *At*IspD (Table 3).

**Table 3:** Overview of the Michaelis Constants (*K*_m_) of Several IspD Homologues

Homologue	*K*_m_^[CTP]^ (μM)	*K*_m_^[MEP]^ (μM)
*Pf*IspD^a^	59 ± 4	61
*Pv*IspD^b^	110 ± 6	/
*Mt*IspD^c^	126 ± 18	92 ± 8
*Ec*IspD^d^	84 ± 9	40 ± 7
*At*IspD^e^	114	500
*Bs*IspD^f^	133 ± 29	125 ± 19

a *Plasmodium falciparum* IspD.^36,37^

b *P. vivax* IspD.^36^

c *Mycobacterium tuberculosis* IspD.^38^

d *Escherichia coli* IspD.^34^

e *Arabidopsis thaliana* IspD.^24^

f *Bacillus subtilis* IspD.^39^

## The Overall Structure of IspD

**Figure 2:**
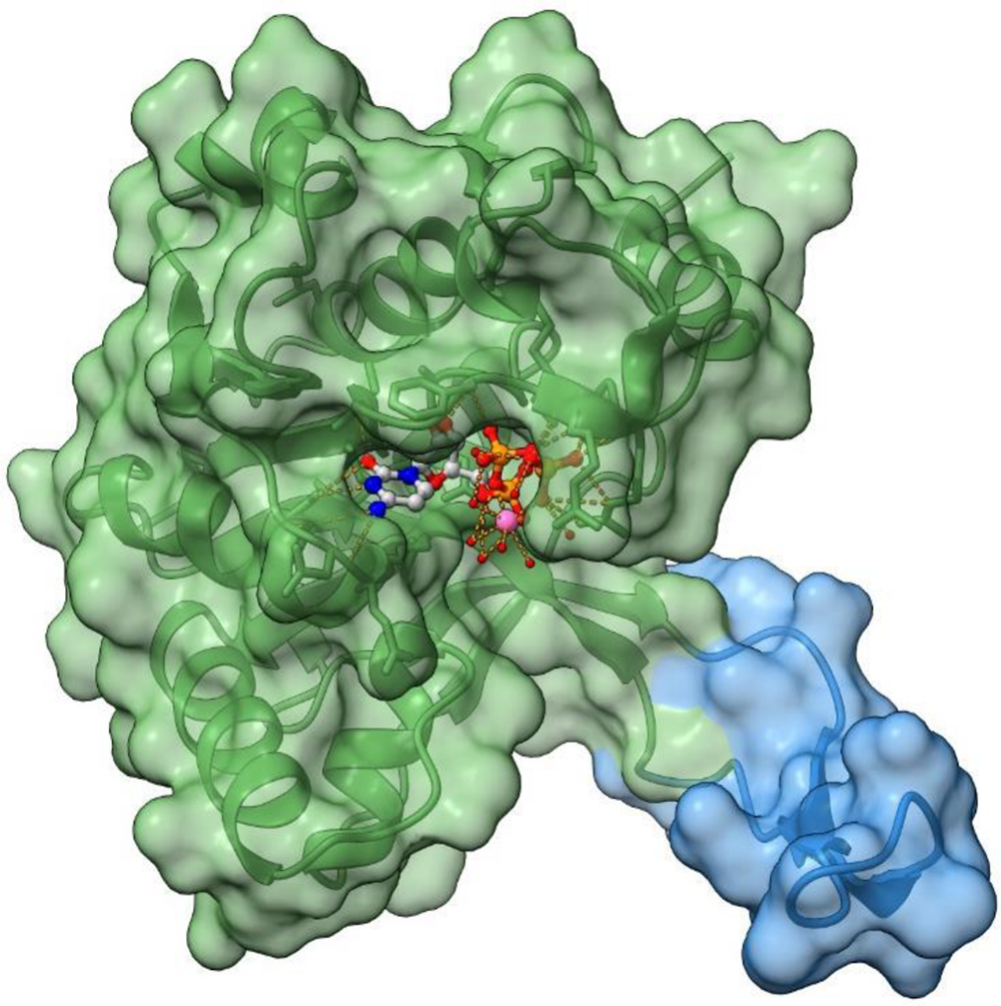
Crystal structure of *Escherichia coli* IspD with CTP in the active site (PDB accession code: 1I52). Visual representation of the two subunits. Green: larger subunit (residues 1–136 and 160–236); Blue: smaller subunit (residues 137–159).

The structure of IspD consists of a homodimer, of which each monomer comprises two structurally different subdomains. The first and largest of these subdomains features an alternating pattern of beta strands and alpha helical segments resembling a Rossmann-like fold with a unique connectivity pattern between both secondary protein structures (Figure 2, green). The Rossmann fold is a tertiary protein structure commonly found in proteins binding nucleotides.^40^ The second, and smaller, subdomain resembles a so-called β-arm, which has a hook-like structure (Figure 2, blue). The subdomain acts as an attachment point to connect both dimers; furthermore, it also plays a significant role in the enzymatic activity as it contains the MEP binding site.^32^ Sequence-based comparison between several pathogenic bacterial species, including *Salmonella typhi*, *Vibrio cholera*, *Haemophilus infuenzae*, *P. aeruginosa*, and *M. tuberculosis*, revealed a high conservation of the overall structure.^41^ Even when monomer structures of *Ec*IspD are compared with its plant-based homologue from *A. thaliana,* significant similarity was observed. However, in this example, care has to be taken as the same comparison between homodimers displays significant differences.^7^ Nevertheless, there is more than one case where the general structure of IspD differs, making drug discovery programs even more challenging. For example, *P. falciparum* IspD (*Pf*IspD) contains over three times more amino acids than its *E. coli* homologue. The structure of *Pf*IspD has not been elucidated to date; however, some homology models have been constructed using *Ec*IspD as a template.^42−44^ Another exception of the general structure is the occasionally observed IspDF, in which, IspD and IspF are covalently linked to one another. The enzyme cluster catalyzes two nonconsecutive steps within the MEP pathway, which is rarely observed for bifunctional proteins. The cluster has comparable activity with its monofunctional counterparts.^46^ Comparison of the active site of crystallized *Campylobacter jejuni* IspDF with *Ec*IspD demonstrated remarkable similarity between both.^47^ Recently, Wu and co-workers elucidated the crystal structure of *Helicobacter pylori* IspDF and found, after comparison, that there was a large similarity with the structure of *C. jejuni* IspDF.^48^

### Active-Site Structure

**Figure 3:**
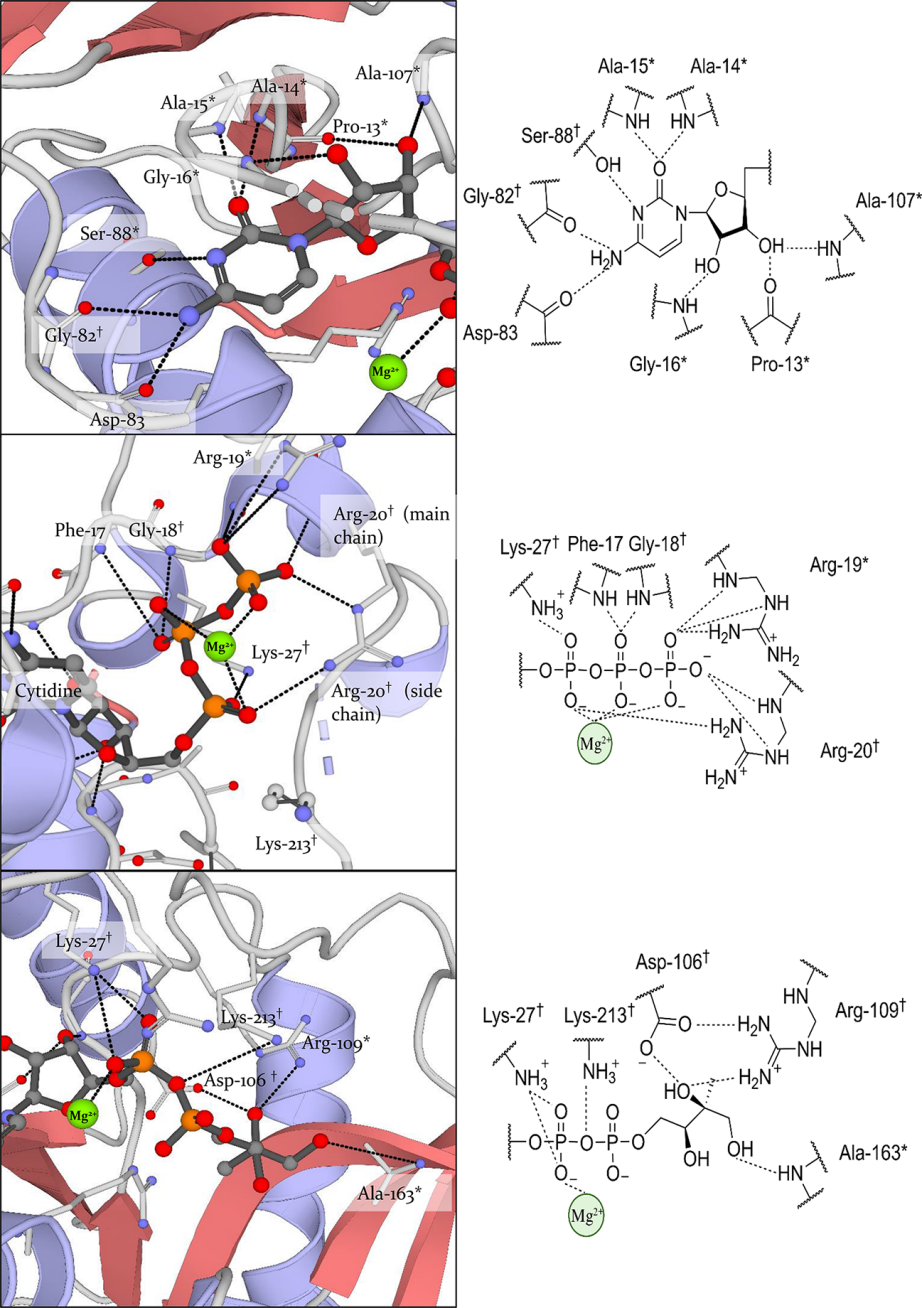
Co-crystal structure of cytidine and CDP-ME in the active site of *Escherichia coli* IspD (PDB accession code: 1INI and 1I52); highly conserved residues (*) present in most IspD homologues; strictly conserved (^†^) present in all IspD homologues. Top: overview of the interactions of the cytidine present in both crystal structures; middle: summary of the interactions of the triphosphate tail of CTP; bottom: interactions of the phosphate and MEP part of CDP-ME. Highly conserved residues (*): Pro-13, Ala-14, Ala-15, Gly-16, Arg-19, Ser-88, Ala-107, Ala-163. Strictly conserved residues (^†^): Gly-18, Arg-20, Lys-27, Gly-82, Asp-106, Arg-109, Lys-213. Not conserved residues: Phe-17, Asp-83.

Insights about the amino acids constituting the active site were obtained from cocrystal structures of *Ec*IspD in complex with both CTP and CDP-ME (PDB accession codes: 1I52 and 1INI, respectively).^32^ When looking at the structures, it becomes apparent that both the cytidine retains the same pocket and its interactions are preserved. In this position, the cytosine occupies a tight pocked formed by hydrogen bonds between Ala-14, Ala-15, Gly-82, Asp-83, and Ser-88 (Figure 3, top). Intriguingly, the size of the pocket accommodating cytosine sterically constrains the use of larger purine bases. The clear preference for cytosine over the other pyrimidine bases is achieved through the specific hydrogen-bond pattern formed between the base and the protein. The ribose, on the other hand, is positioned in a more open space in which one of the hydroxy groups forms hydrogen bonds with the backbone carbonyl of Pro-13 and backbone amide Ala-107, while the other hydroxyl forms a hydrogen-bond with the backbone amide of Gly-16 (Figure 3, top). The major difference between both structures is found in the interactions formed by the phosphates of CTP and CDP-ME. The phosphates of CTP are stabilized by coordination with the Mg^2+^ ion. Both α- and γ-phosphates are involded in a hydrogen bonding network with Arg-20. Furthermore, the α-phosphate is in direct contact with Lys-27. These interactions play a key role in the presumed mechanism, as they prime CTP for nucleophilic attack by MEP and stabilize the postulated pentacoordinate transition state (Figure 3, middle). The involved residues were found to be conserved in several pathogenic bacterial species including *S. typhi*, *V. cholera*, *H. infuenzae*, *P. aeruginosa,* and *M. tuberculosis*, emphasizing their importance. When shifting the focus to the cocrystal structure of CDP-ME (PDB accession code: 1INI), it becomes apparent that most interactions formed between the phosphates of CTP and the protein diminish when CDP-ME is formed (Figure 3, bottom). This, in turn, facilitates the release of the product. The interactions formed between MEP and the protein are more difficult to trace, as no cocrystal structure of IspD in complex with MEP has been solved to date. An explanation for this could be the proposed sequential mechanism by which CTP should bind to IspD before MEP could bind. Based on the cocrystal structure of IspD in complex with CDP-ME, MEP seems to form hydrogen bonds with Arg-157 and Lys-213 (Figure 3, bottom).^32^ All of these highly polar interactions make the IspD active site the most polar among all MEP pathway enzymes. Later reported crystal structures of *At*IspD (PDB accession code: 1W77),^7^*M. tuberculosis* IspD (*Mt*IspD) (PDB accession code:2XWN),^45^*M. smegmatis* IspD (*Ms*IspD) (PDB accession code: 2XWM),^45^ and *B. subtilis* IspD (*Bs*IspD) (PDB accession code *apo* structure: 5DDT, CTP-Mg^2+^ complex: 5HS2)^39^ demonstrated highly similar active sites. As with the general structure, a sequence comparison between various pathogenic bacteria demonstrated great conservation of the amino acids lining the active site of IspD, even for *Pf*IspD.

### Allosteric Pocket

Besides the active site, IspD is also believed to have a targetable allosteric pocket in close proximity of the active site. This pocket is rather flexible, is not visible in the *apo*-protein, and only opens upon binding of an inhibitor. At this point in time, only inhibitors for *At*IspD have been confirmed targeting this pocket, but it is assumed that it is also present in most other IspD homologues.^12,14,49,50^ The pocket in *At*IspD consists of the following amino acids: Leu-45, Arg-157, Val-161, Ala-202, Val-204, Gln-238, Val-239, Ile-240, Phe-249, Asp-262, Ser-264, Ile-265, Val-266, and Val-273. Taking this into account, we can see that it is much less hydrophilic than the active site, making it more appealing for future drug development. Initial experimentation toward the elucidation of the biological role of the allosteric pocket was performed by Schwab *et al*.^49^ They found that the allosteric pocket is not involved in a feedback loop with either DMADP or IDP, both of which are weak inhibitors targeting other regions of the enzyme. Until now, the biological function of the allosteric pocket remains unknown.

## Human IspD

Despite the absence of the MEP pathway in humans, recent research suggests that the human homologue of IspD (*Hs*IspD) is an essential enzyme for dystroglycan *O*-mannosylation. Defects or deficiency in dystroglycan *O*-mannosylation lead to muscular dystrophy, severe brain abnormalities, and, in some cases, the Walker–Warburg syndrome.^51,52^ Analysis of the crystal structure of *Hs*IspD demonstrated strong similarity with the active site of *Ec*IspD, and, in extension, the whole larger subdomain. Significantly less conservation is observed for the small subdomain; especially, the sequential motifs are completely different. At this point in time, the functional activity of *Hs*IspD remains unclear. Furthermore, it is unknown where the enzymatic reaction of *Hs*IspD takes place: this could be either in the active site or in a newly formed active site on the smaller subdomain.^53^ Taking this into account, off-target inhibition of *Hs*IspD could pose a serious caveat against the development of IspD inhibitors for medicinal purposes. Despite this, we would advise for a case-to-case evaluation of the activity toward *Hs*IspD by the inhibitor. For example, it was seen that inhibition of pathogenic IspD does not automatically lead to inhibition of *Hs*IspD. Ghavami *et al*. demonstrated that **MMV-008138**, a potent inhibitor of *Pf*IspD targeting the CTP binding site, does not inhibit the active site of *Hs*IspD.^54^

## Strategies to Identify IspD Inhibitors

Three distinct methodologies have been employed to investigate the potential IspD inhibitors. The most commonly used method employs the use of an enzymatic assay to test large compound libraries on their ability to inhibit a certain homologue of IspD.^43,55,56^ The main advantage of this method is the ability to screen large libraries of both small molecules and fragments quickly and straightforwardly.

In the second method, a rescue assay is employed. In this experimental setup, the target organism is cultivated in two distinct media. Both of these media contain the potential inhibitor being tested; however, one of the media also includes IDP. If the potential inhibitor specifically targets the MEP pathway, the expectation is that growth inhibition will occur in the medium that lacks IDP. In contrast, in the medium that is supplemented with IDP, growth inhibition should be either absent or only partial.^57^ Screenings carried out in this way will result in selective inhibitors for the MEP pathway that are able to penetrate and reach the target site. Despite the clear advantage in selectivity for MEP pathway enzymes, these screenings are work-intensive and require the cultivation of potentially dangerous pathogens. In addition, further research is needed to pinpoint the targeted enzyme within the MEP pathway.

Lastly, modification of one of the substrates can also yield potential inhibitors. This technique is mostly limited to MEP due to the widespread occurrence of CTP.^58^ Unfortunately, MEP mimics have an elaborate synthesis due to their high polarity, phosphate group, and the many chiral centers.^34,59−61^

Ideally, a combination of the first two techniques is used in succession to benefit first from the swiftness of the enzymatic assay, and later from the selectivity of the rescue assay to determine the most ideal hit for further optimization. To facilitate this process, one can always use virtual techniques to filter compound libraries and to select the most interesting compounds to screen.^62^ Molecular-docking studies can also be used to have some preliminary filtering of compound libraries.

## IspD Inhibitors

Based on the crystal structures just described, it is clear that IspD constitutes a rather challenging target due to the high polarity, solvent exposure, and flexibility of the protein. Nevertheless, many groups focused on the discovery of selective IspD inhibitors over the years because it is an attractive target to combat AMR. Nonetheless, there is still a lack of IspD inhibitors that efficiently target the various homologues and feature good whole-cell activity as well as pharmacokinetic properties. Fortunately, the reported IspD inhibitors display a wide range of modes of inhibition (MOIs), which will inspire the design of future inhibitors. We will present the inhibitors categorized based on their MOI.

### Competitive Inhibitors

Within the following paragraphs, we will discuss all IspD inhibitors that target the active site of IspD. Throughout the years, inhibitors have been found that compete with either MEP or CTP.

#### BITZ Chemical Class

**Figure 4:**
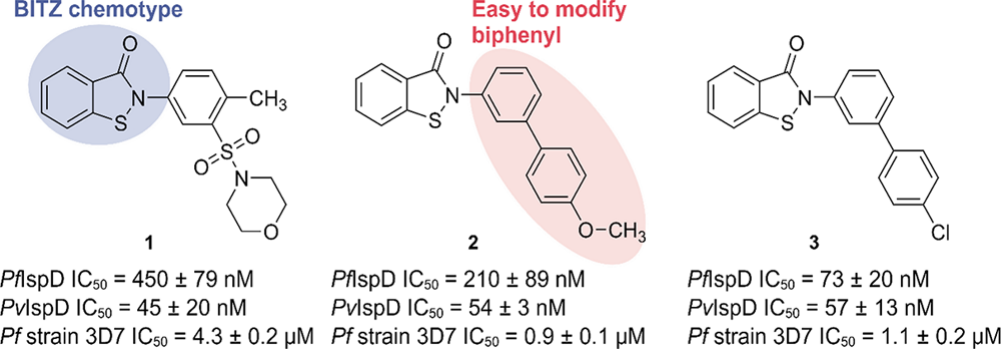
Overview of 2-phenyl benzo[*d*]isothiazol-3(2*H*)-one (BITZ) compounds and their IC_50_ values against *Plasmodium falciparum* (*Pf*) and *P. vivax* (*Pv*) IspD, and growth inhibition against the *Pf* strain 3D7.

**Scheme 3:**
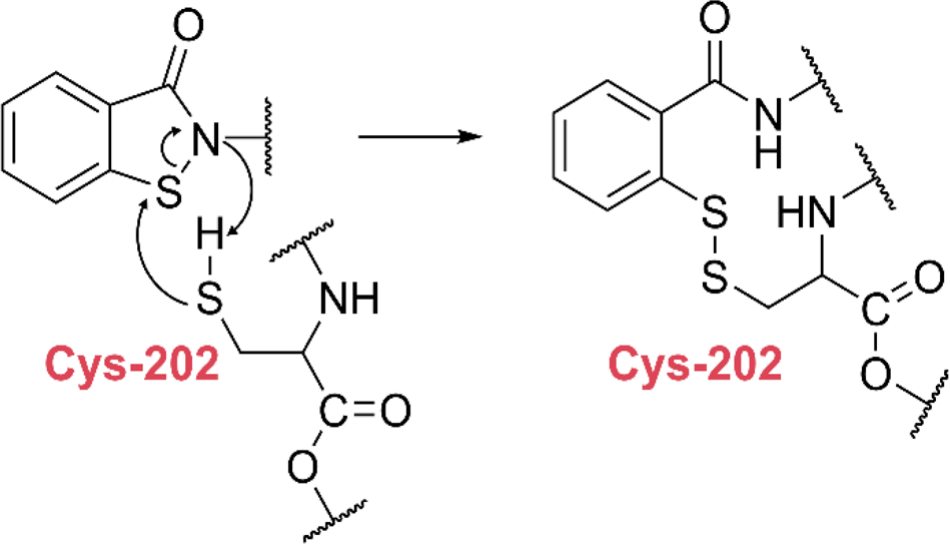
Presumed Covalent Mechanism of 2-phenyl benzo[*d*]isothiazol-3(2*H*)-one (BITZ)

By a combined approach of cheminformatics and high-throughput enzymatic screening, Hale and co-workers discovered a sub-micromolar *Pf*IspD inhibitor starting from a commercial compound library of 500,000 compounds (BioFocus DPI).^62^ During the workflow, similarity searches and scaffold-hopping were used to isolate interesting compounds from the library, resulting in a selection of around 10,000 compounds that were experimentally screened against *Pf*IspD. During this screening, the 2-phenyl benzo[*d*]isothiazol-3(2*H*)-one (BITZ) chemotype was repeatedly noticed. Initial hit **1** exhibited an IC_50_ value of 450 ± 79 nM on *Pf*IspD and 45 ± 20 nM on *P. vivax* (*Pv*) IspD (Figure 4). Additionally, **1** also demonstrated low-micromolar activity (Strain 3D7 EC_50_ = 4.3 ± 0.2 μM) in a whole-cell assay. A follow-up SAR was performed for the BITZ chemotype. As the first step in the SAR, the sulfonyl-morpholine was replaced by a biphenyl group, allowing for easier modifications on both rings (Figure 4). Optimization led to compound **3**, for which potency was enhanced against *Pf*IspD (IC_50_ = 73 ± 20 nM) while retaining low nanomolar activity on *Pv*IspD. As a result, the two homologues likely feature a similar binding pocket. Besides good enzymatic activity, BITZ compounds also exhibit growth inhibition against the 3D7 strain of *P. falciparum* with EC_50_ values ranging from low micromolar to high nanomolar (Figure 4). Furthermore, when **2** was tested against several *P. falciparum* strains resistant to the most commonly used malaria treatments, similar results were obtained (Table 4). To verify that the growth inhibition is a result of tackling the MEP pathway, and in particular IspD, early ring-stage cultures of *P. falciparum* were treated with **2** at five times the IC_50_ value. Metabolic profiling of these cultures revealed a significant decrease in downstream MEP metabolites and normal levels of upstream metabolites, hence validating IspD as a target. Despite these encouraging results, it became apparent that IspD was not the only target, as IDP supplementation during rescue experiments did not lead to survival of the parasite. To elucidate the binding mode of this compound class, a homology model was constructed based on a previously published *Ec*IspD crystal structure (PDB accession code: 1I52).^32^ Docking of compound **2** into the homology model afforded the best result when **2** was present inside the CTP binding site. In this pose, the isothiazolidin-3-one moiety was observed to be in the proximity of Cys-202. This observation led to the proposal that BITZ operates through a covalent mechanism wherein the thiol of Cys-202 reacts with the sulfur atom in the isothiazolidin-3-one moiety, leading to ring opening and affording a disulfide bond between IspD and BITZ (Scheme 3). To confirm their proposal, both time- and dose-dependent inhibition kinetics were studied. Results from both experiments pointed toward a covalent inhibition mechanism. To further corroborate this hypothesis, a mutant was constructed, wherein Cys-202 was replaced with an alanine. This replacement led to a 6-fold decrease in sensitivity of **2** (*Pf*IspD-[Cys202Ala] IC_50_ = 470 ± 39 nM *vs Pf*IspD IC_50_ = 210 ± 89 nM). Lastly, the BITZ compounds proved inactive against *Ec*IspD, in which Cys-202 is not present.

**Table 4:** Assessment of **2** against Various Resistant *Plasmodium falciparum* Strains

*Plasmodium falciparum* strain	Whole-cell growth inhibition EC_50_ (μM)
3D7 (control)	0.9 ± 0.1
D6 (mefloquine-resistant)	0.8 ± 0.1
7G8 (chloroquine-resistant)	1.0 ± 0.3
IPC 5202 (artemisinin-resistant	1.4 ± 0.2

#### MMV-08138

**Figure 5:**
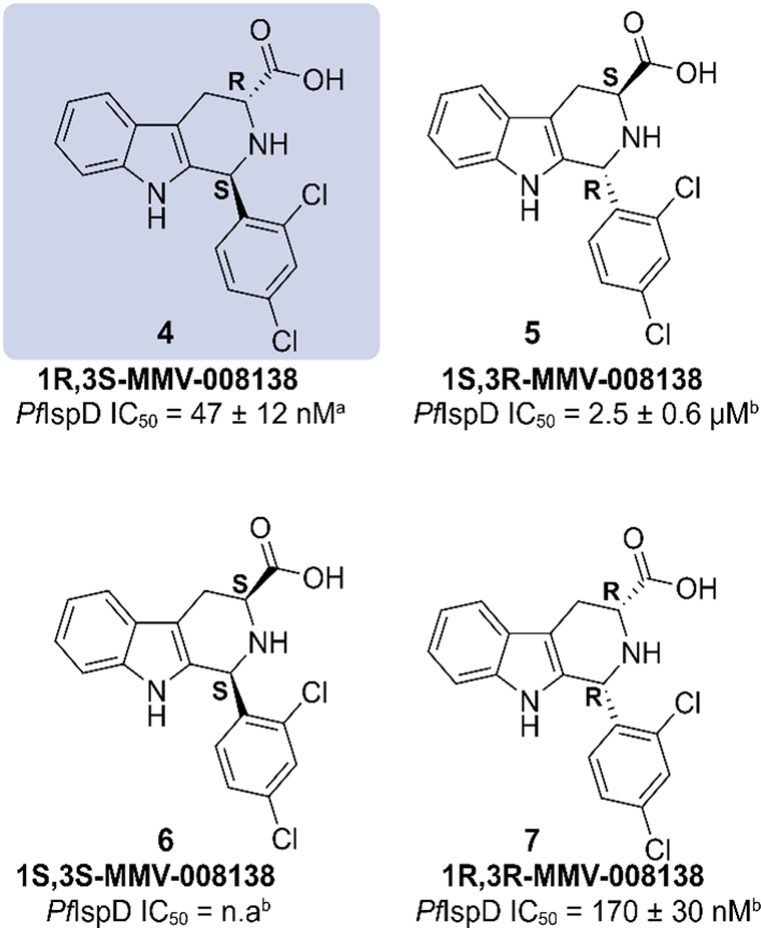
Overview of the enantiomers of **MMV-008138** and their IC_50_’s against *Plasmodium falciparum* (*Pf*) IspD; ^a^ref (36); ^b^ref (37).

**Figure 6:**
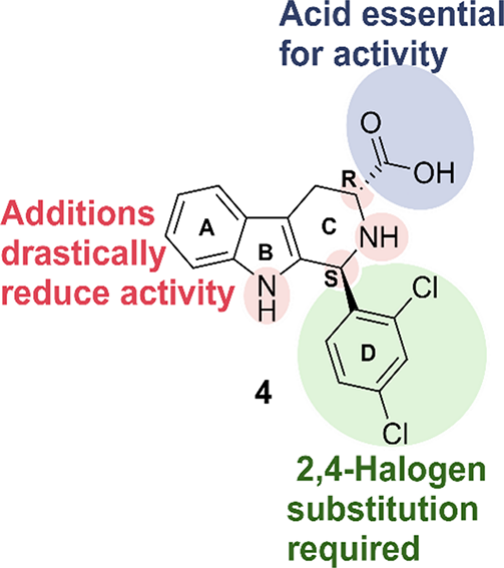
Overview of the structural features key for the activity of the **MMV-008138** chemotype.

**Figure 7:**
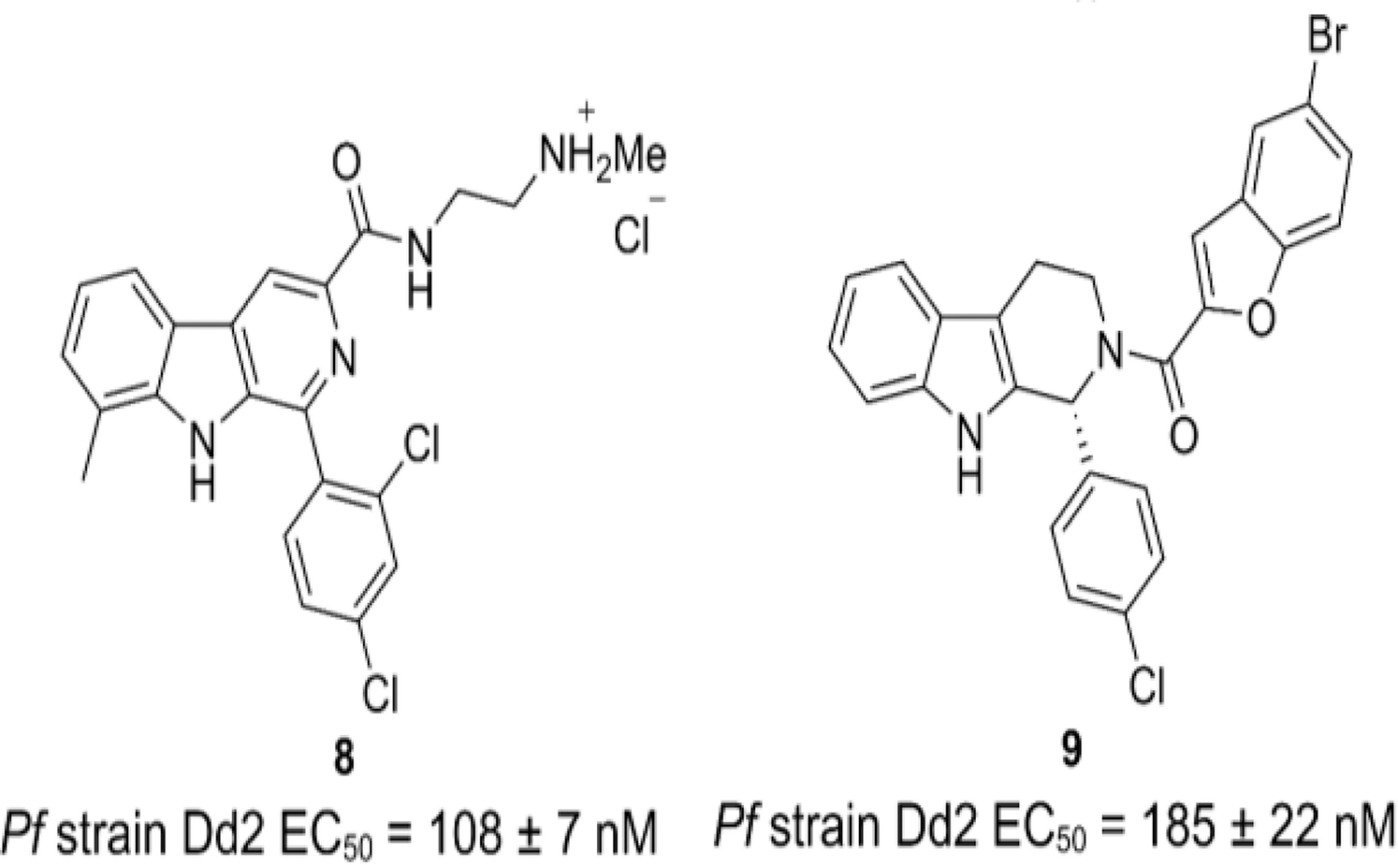
New chemical classes derived from the **MMV-008138** chemotype demonstrating promising growth inhibition of *Plasmodium falciparum* (*Pf*) strain Dd2; **8** ref (66); **9** ref (67).

By far, the most explored IspD inhibitor to date is **MMV-08138**, which was identified through a phenotypic IDP rescue screening of the malaria box compounds (Figure 5). The screening was performed in this way to ensure hit selectivity for the MEP pathway enzymes. **MMV-08138** proved to be capable of inhibiting 95% of growth at 5 μM when IDP was absent from the medium; on the contrary, IDP supplementation compromised the majority of the growth inhibition.^57^ During screening efforts, the racemic mixture was used, and only after enantiomeric separation the activity could be attributed to the 1*R*,3*S* conformer (**4**) (*Pf*IspD IC_50_ = 47 ± 12 nM, Figure 5). Interestingly, the other conformers were either significantly less or even inactive. On account of the phenotypic character of the screening, the targeted enzyme within the MEP pathway had to be elucidated before optimization could commence. To do so, the authors generated **MMV-08138**-resistant strains by exposing susceptible parasites to the inhibitor, until a resistant strain emerged. This experiment was repeated three times, once with the racemic mixture of **MMV-08138** at lethal dose, once with the 1*R*,3*S* conformer (**4**) at IC_75_ concentration, and lastly the resistant strain grown at IC_75_ concentration was exposed to a lethal dose. Next, the whole genome of the three resistant strains was sequenced and compared to those of the parent and reference strains. This comparison gave rise to the discovery of a mutation in the gene encoding IspD for all three resistant strains. Two unique mutations were identified; the first being an exchange from glutamate to glutamine at position 688 [Glu688Gln]; the second a conversion of a leucine to isoleucine at position 244 [Leu244Ile].^37^ To fully confirm that these modifications were responsible for the resistance toward **4**, both IspD variants were expressed and purified. Activity determination of **4** against the purified enzymes led to IC_50_ values of 100 ± 24 nM for *Pf*IspD-[Glu688Gln] and 320 ± 165 nM for *Pf*IspD-[Leu244Ile], compared to an IC_50_ value of 47 ± 12 nM for wild-type IspD. Additional metabolic profiling of *P. falciparum* parasites, treated with **4**, displayed significantly reduced levels of downstream MEP pathway metabolites in comparison with control parasites and parasites treated with chloroquine or artemisinin.^36^ Both findings consolidate inhibition of IspD as the MOI. With the target known, the binding mode could be elucidated by determining the enzymatic kinetics at varying substrate and inhibitor concentrations. Analysis pointed in the direction of noncompetitive and competitive inhibition toward MEP and CTP, respectively. This finding suggests that **4** binds within the CTP binding pocket. To get an idea of which interactions might play a role, **4** was docked into the active side of a homology model based on the *Ec*IspD structure (PDB accession code: 1I52). The prediction showed that an array of four hydrogen bonds between the carboxylic acid and Thr-664, Arg-208, and Lys-215 would presumably be the key interaction. Lastly, the spectrum of **4** was explored against those of other IspD homologues. Interestingly, **4** demonstrated specific inhibition for *Plasmodial* species with great activity toward *Pv*IspD (IC_50_ = 310 ± 80 nM), while being inactive toward both *Ec*IspD and *Mt*IspD.^36^ Until today, multiple SAR studies have been conducted on the **MMV-0008138** chemotype, although none of them led to an improvement in potency. Nonetheless, these studies exposed some of the structural features that are key for the activity. For example, a 2,4-halogen substitution pattern was observed at the d-ring and the carboxyl group, although replacement with methyl amide retained most of the activity (Figure 6). Furthermore, it was seen that any extensions to the nitrogen atoms or chiral centers led to a complete loss of activity, presumably due to conformational changes imposed by additions at these sites (Figure 6).^63−65^ Due to the promising nature of the **MMV-0008138** chemotype, several structure-similarity searches have been run on the libraries constituting the Malaria Box. This led to the discovery of two new classes, closely resembling **MMV-0008138**, with promising antimalarial activities. Unfortunately, IDP rescue assays conducted for both new classes revealed that IspD is no longer the primary target for these new classes (**8**, **9**; Figure 7).^66,67^

#### MEPN_3_

**Figure 8:**
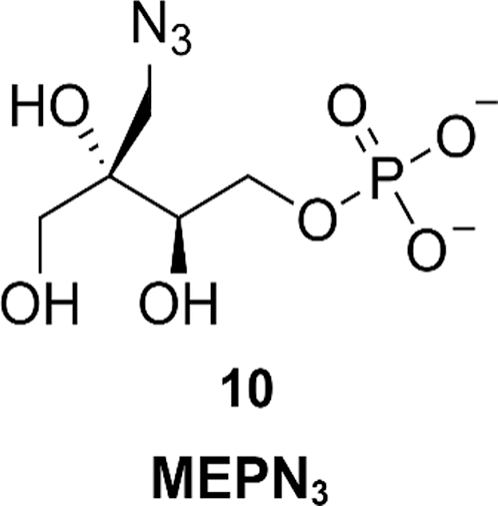
MEPN_3_ analogue of 2-*C*-methyl-d-erythritol 4-phosphate (MEP).

With the objective to design an IspD inhibitor suitable for fragment-based drug discovery approaches, Baatarkhuu *et al*. reasoned that the introduction of an azide moiety in MEP would give rise to an interesting starting point.^34^ With this idea in mind, the introduction of the azide handle was performed at the 2*C*-methylene, to not interfere with any of the hydroxyl groups involved in hydrogen bonds. The resulting compound (**10**, Figure 8), named MEPN_3_, exhibits an IC_50_ value of 41.5 ± 3.8 μM against *Ec*IspD. Determination of the MOI of this new inhibitor was done with steady-state inhibition kinetics. This led to the discovery that **10** is able to bind to both substrate binding sites, albeit with a preference for the MEP binding pocket. Furthermore, **10** favors the free enzyme above the enzyme–substrate complex, while the opposite is true for MEP. Docking studies performed on a cocrystal structure of *Ec*IspD in complex with CDP-ME (PDB accession code: 1I52) further substantiated this MOI. The docking study also revealed enough space within the binding pocket to extend **10** with the formation of a triazole, which would allow further optimization of this compound class, which is yet to be reported.

### Allosteric Inhibitors of IspD

**Figure 9:**
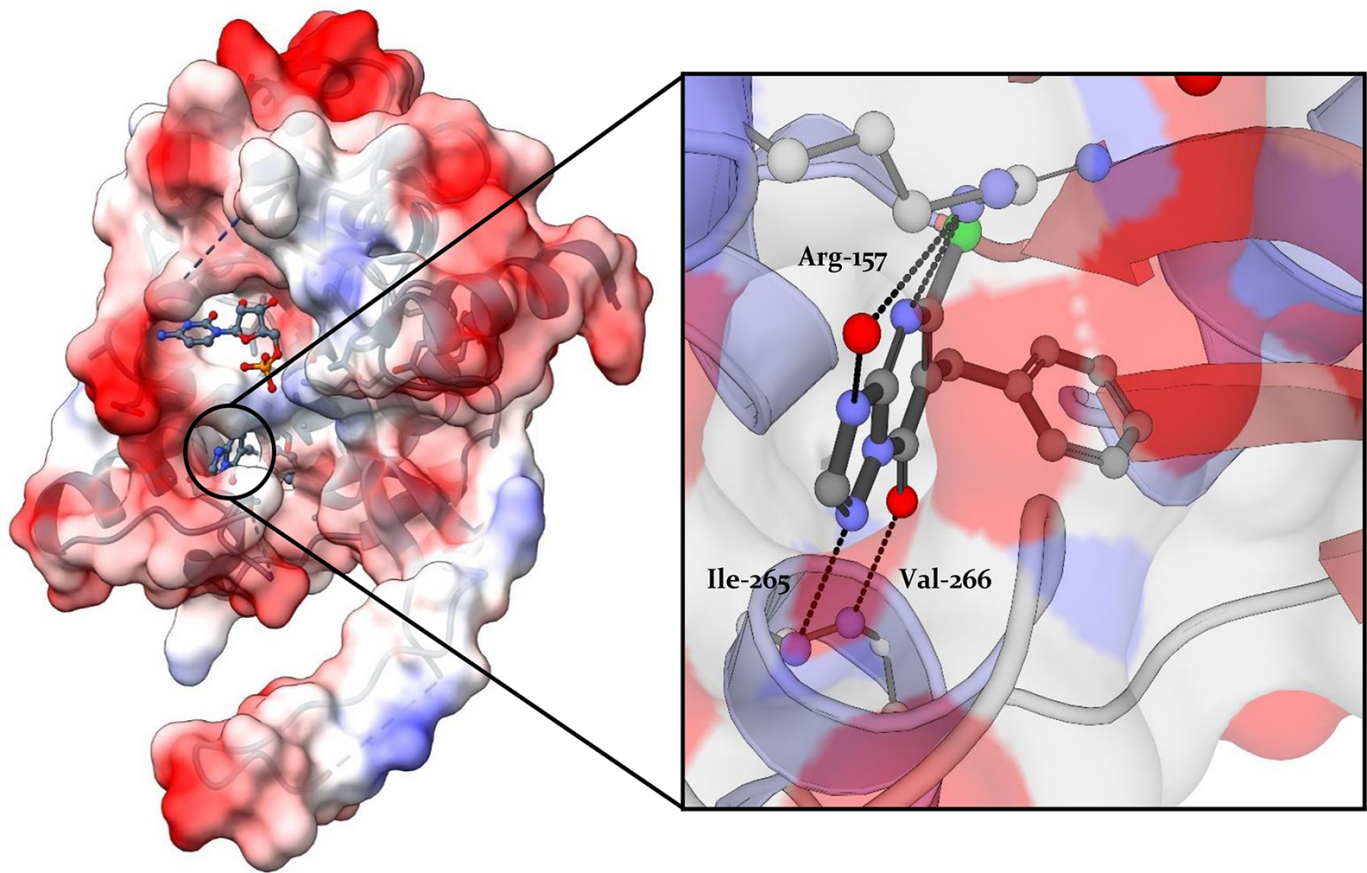
Crystal structure of *Arabidopsis thaliana* IspD with **11** in the allosteric pocket displaying the key interactions, which **11** is engaging with the allosteric pocket (PDB accession code: 2YC3).^14^ The allosteric pocket consists of the following residues: Leu-45, Arg-157, Val-161, Ala-202, Val-204, Gln-238, Val-239, Ile-240, Phe-249, Asp-262, Val-263 Ser-264, Ile-265, Val-266, and Val-273.

As mentioned before, IspD features an allosteric site with a more favorable lipophilic character (Figure 9). At the moment, all reported allosteric IspD inhibitors target *At*IspD.

#### Azolopyridines

**Figure 10:**
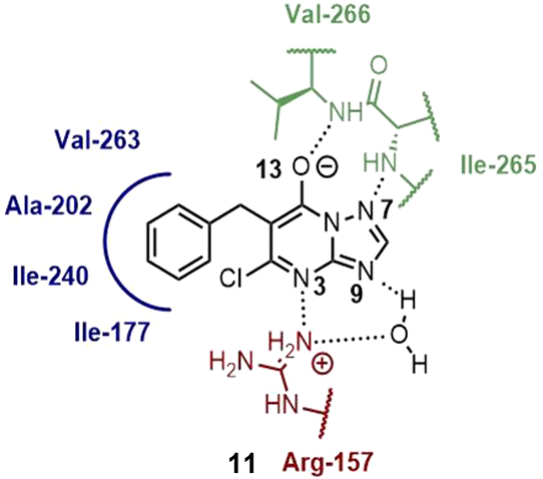
Initial hit (**11**) of the azolopyridine class with the points of interaction in the allosteric pocket highlighted.^14^

**Figure 11:**
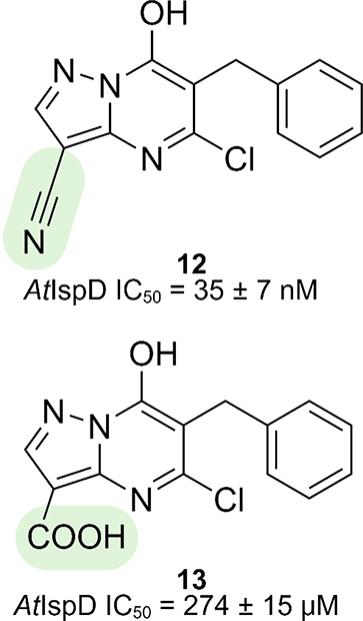
Derivatives of the azolopyridine class designed to displace a water molecule inside the allosteric pocket.^14^

Azolopyridines were discovered during a HTS of over 100,000 compounds, to find compounds targeting *At*IspD at BASF. **11**, endowed with an IC_50_ value of 140 ± 10 nM against *At*IspD, caught the interest of Witschel *et al*. Elucidation of the cocrystal structure of *At*IspD in complex with **11** (PDB accession code: 2YC3) uncovered that the compound occupies the allosteric pocket instead of the active site (Figure 9 and 10). Within this allosteric pocket, the phenyl ring fits tightly into a hydrophobic subpocket, in which it makes a multitude of lipophilic interactions. In addition, **11** forms four hydrogen bonds: between N3 and Arg-157, between the deprotonated hydroxyl and the backbone NH of Val-266, between N9 and Ile-265, and lastly between N7 and a highly localized water molecule at the entrance of the pocket. The authors tried to enhance the potency by displacing this highly ordered water molecule at the entrance of the allosteric pocket through the addition of a nitrile or carboxylic acid at the N9 position, respectively (Figure 10). Both moieties succeeded in displacing the water molecule and, by doing so, gained an extra hydrogen bond with the protein (PDB accession code: 2YC5, nitrile; 2YMC, carboxylic acid; Figure 11). Despite this, only in the case of the nitrile an increase in potency (**12**, *At*IspD IC_50_ = 35 ± 7 nM) was observed. In the case of carboxylic acid (**13**, *At*IspD IC_50_ = 274 ± 15 μM), the gain in potency is negligible in comparison with the energy cost needed for the desolvation of the carboxylate moiety upon binding. Further modifications were directed at the phenyl ring, but even the smallest increase in volume led to reduced activity.^14^ A more detailed description of the allosteric MOI can be found below. Further development of the *in vivo* herbicidal activity of the azolopyridines was performed by Clough *et al.*, obtaining promising results, although it became apparent that IspD was no longer the *in vivo* target of these new derivatives.^68^

#### Pseudilines and Derived Compound Classes

**Figure 12:**
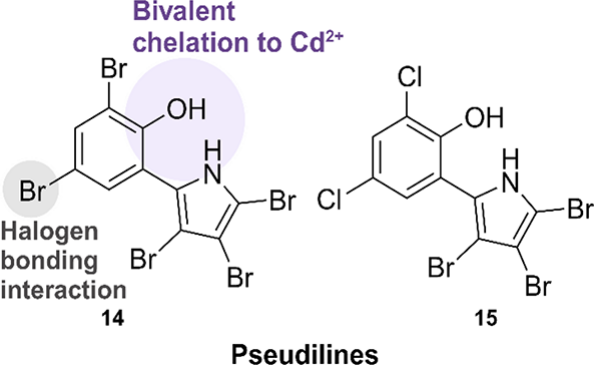
Summary of the interactions between the pseudilines, *Arabidopsis thaliana* IspD, and Cd_2_^+^.^50^

The following compound class contains four “generations” of hit molecules discovered and optimized by different research groups. The main structural motif of this class is a direct link between a highly halogenated phenyl ring and a 5-membered heterocycle (Figure 12). The first generation of this class was discovered by BASF during a HTS-campaign using a spectrophotometric *At*IspD inhibtion assay.^50^ This led to the discovery of several hits exhibiting IC_50_ values below 25 μM, among which pseudilines **14** (*At*IspD IC_50_ = 13 ± 2 μM) and **15** (*At*IspD IC_50_ = 12 ± 1 μM) displayed the best activity (Figure 12). Pseudilines are a class of highly halogenated natural products with antibiotic properties originally isolated from seawater-derived bacteria in 1966 by Burkholder and co-workers.^69,70^ A handful of additional pseudiline derivatives were synthesized, but despite these efforts, no improvement in activity was achieved. A cocrystal structure was acquired, from which it became apparent that the pseudilines bind in the same allosteric pocket as the azolopyridines, demonstrating the flexible character of this pocket (PDB accession code: 4NAL, **14**; 4NAN, **15**). The main interaction seen in the binding pocket is a bivalent chelation of the pseudilin hydroxyl and pyrrole nitrogen atoms to a Cd^2+^ cation. The tetrahedral coordination of the Cd^2+^ cation is completed by interactions with the Gln-238 side chain and a water molecule present in the binding pocket. The presence of Cd^2+^ in the crystal structure results from the use of CdSO_4_ during crystallization. Bivalent metal ions are frequently added to protein crystallization conditions to promote crystallization.^71^ Further interactions with the protein include a halogen-bonding interaction between the halogen atom in the *para* position of the hydroxyl and the carbonyl oxygen atom of Val-239. The interactions formed with Cd^2+^ cation in the crystal structure prompted Kunfermann *et al*. to repeat activity measurements in the presence of 40 μM of CdSO_4_. This addition led to a 10-fold increase in the activity for both hits (Table 5). Further profiling of the pseudilines led to the detection of micromolar activity against *Pv*IspD.

**Table 5:** Summary of the Activity of the Pseudilines^a^

	*At*IspD IC_50_ (μM)		*Pv*IspD IC_50_ (μM)	
*#*	W/O CdSO_4_	40 μM CdSO_4_	W/O CdSO_4_	40 μM CdSO_4_
**14**	13 ± 2	1.4 ± 0.2	48 ± 9	57 ± 12
**15**	12 ± 1	2.2 ± 0.2	56 ± 8	41 ± 7

a Data shown for both *Arabidopsis thaliana* IspD and *Plasmodium vivax* IspD without and within the presence of 40 μM CdSO_4_.

**Figure 13:**
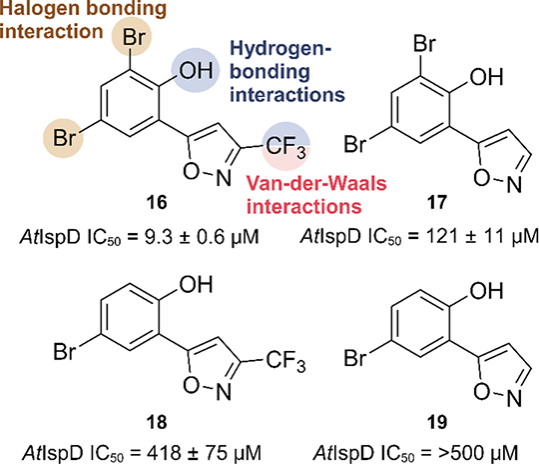
Chemical structure of compounds **16-18**, their activity against *At*IspD and summary of the interactions with *Arabidopsis thaliana* IspD.

A second screening, also performed at BASF, with compounds having similar chemical structure as pseudilins **14** and **15** gave rise to the discovery of compound **16** (*At*IspD IC_50_ = 9.3 ± 0.6 μM) (Figure 13).^49^ While the general scaffold remains similar, the pyrrole is replaced by an isoxazole, and consequently, the ability to make bivalent coordination was lost and hence also the benefit from the addition of bivalent metal ions. The authors again reverted to cocrystallization to elucidate the MOI (PDB accession code: 5MRM). Similar to the pseudilins, the halogen in the *para* position forms a halogen-bonding interaction with the carbonyl oxygen of Val-239. In this case, the halogen in the *ortho* position is also capable of forming a halogen bond with the carbonyl oxygen atom of Glu-267 (Figure 13). This interaction proved to be essential, as replacement of the halogen by hydrogen resulted in a 10-fold loss in activity. The hydroxyl forms hydrogen-bonding interactions with the side chain hydroxyl group of Ser-264 and an ordered water molecule present in the pocket. The CF_3_ moiety on the isoxazole ring is positioned in a small pocket, in which it forms a multitude of interactions, contributing greatly to the activity. Lastly, some π-stacking interactions of the phenyl ring with the methyl groups of Val-266 and the carboxyl side chain of Gln-238 were seen to contribute to the overall affinity of the compound.

Interestingly, although the azolopyridines, pseudilines, and the isoxazoles bind in the same allosteric pocket, different conformational changes are imposed by the different classes, which, in turn, lead to unique allosteric mechanisms. For example, binding of compound **11** or its derivatives leads to a protrusion of Asp-262 into the MEP pocket, preventing MEP from binding. While engagement of pseudilins blocks the CTP ribose binding site by displacement of Arg-157, it also causes steric and electrostatic repulsions in the MEP binding pocket by displacement of Asp-261. The allosteric mechanism of the isoxazoles is to a degree in between both mechanisms; Arg-157 stays in place, while both Asp-261 and Asp-262 protrude into the active site and cause electrostatic repulsions, hindering both substrates from binding. A complete overview of all residues affected by binding of the inhibitors inside the allosteric pocket, as well as graphical representation, can be found elsewhere.^49^

Interestingly, Wang *et al*. tried to further enhance the activity by replacing the isoxazole ring by a pyrazole.^72^ A wide variety of substitution patterns with this scaffold was explored, although this did not lead to any significant improvement over the isoxazole. Docking studies using the *At*IspD-isoxazole complex (PDB accession code: 5MRM)^49^ as a template revealed that this new class has similar interactions with the protein as the isoxazoles. Furthermore, predictions show that the NH does not participate in any interaction with the protein.

Lastly, Zhang and co-workers combined the scaffolds of **14** and Diuron, a commercial herbicide, to further expand the scope of the pseudilines toward algae.^73^ Ultimately their efforts resulted in several compounds exhibiting promising anticyanobacterial activity, of which one compound also demonstrates moderate inhibition of *Ec*IspD (91% inhibition at 100 μM). These derivatives display very low or even no inhibitory activity toward *At*IspD.

### Inhibitors with Unknown MOI

In the following, we highlight IspD inhibitors for which the MOI is still unknown.

#### Pyrrolopyrazines

**Figure 14:**
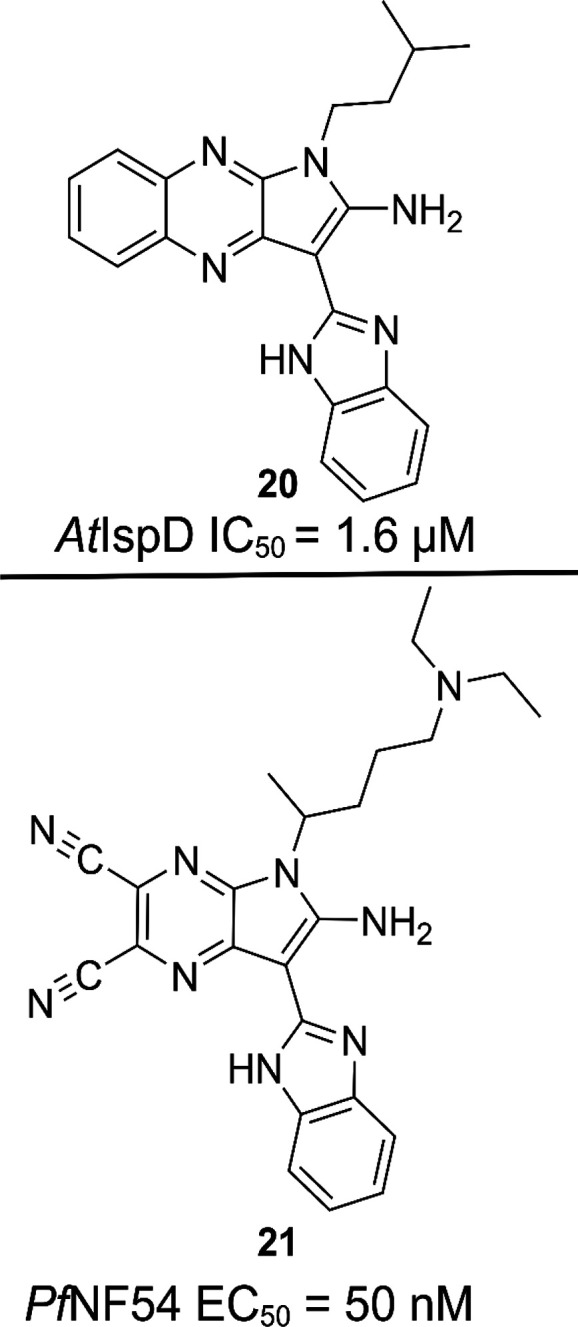
Top: initial pyrrolopyrazine, *Arabidopsis thaliana* (*At*); bottom: pyrrolopyrazine derivatives, *Plasmodium falciparum* (*Pf*).^74^

The first class of compounds discussed here are the pyrrolopyrazines.^74^ This class was discovered at BASF during a HTS aiming to find inhibitors of *At*IspD. The initial hit **20** was found to have an IC_50_ value of 1.6 μM for *At*IspD (Figure 14). Many derivatives were synthesized and tested, but none showed any improvements over **20**. The compound class was later tested against asexual blood stages of the *Pf*NF54 strain, wherein it showed excellent potency (EC_50_ ≈ 200 nM). Further optimization led to an array of analogues with low nanomolar activity on *Pf*NF54, while also featuring good selectivity and low toxicity. In addition, their lead compound **21** demonstrated excellent activity against liver schizont stage in the *P. berghei* mouse model. The discrepancy in activity between *At* and *Pf* led the authors to believe that there are additional or different enzymes being targeted by the pyrrolopyrazines. A wide array of computational studies were performed to elucidate those targets, which ended up pointing toward kinases, and more specifically, *Pf*PK5. Inhibitory activity of the lead compound for this enzyme was seen at a concentration of 30 μM, but further experiments have to be performed to unambiguously assign *Pf*PK5 as the cellular target of the pyrrolopyrazines.

#### Urea Class

**Figure 15:**
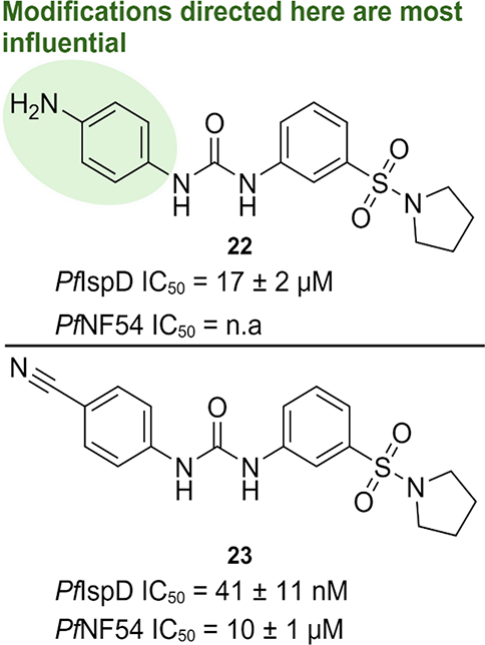
Top: initial urea hit; bottom: promising urea compound. Activities against both *Plasmodium falciparum* (*Pf*) IspD and *Pf*NF54 strain are displayed.^75^

The next class was discovered during an HTS campaign targeting *Pv*IspD after which all hits were concomitantly tested against *Pf*IspD and *Pf*NF54 cells.^75^ The initial hit compound **22** had an IC_50_ value of 17 ± 2 μM on *Pf*IspD but was lacking whole-cell activity. We next optimized the activity of the compound class with a focus on retaining the straightforward synthesizable urea linker. The SAR study resulted in a 400-fold increase in the activity on *Pf*IspD and activity in a whole-cell assay (Figure 15). During the SAR study, we noticed that modifications directed at the Western ring (circled in green in Figure 15) had the highest impact on the potency. Especially, electron-withdrawing moieties at the *para* position resulted in increased potency. With the optimized compounds in hand, we tried to elucidate the MOI by kinetic characterization in the presence of different inhibitor concentrations. We noticed that compound **23** was not competing with any of the substrates, hinting toward allosteric inhibition. Follow-up experiments are required to confirm this hypothesis, but this could imply that the urea class would be the first inhibitor targeting the allosteric pocket of an IspD homologue other than *At*IspD. Lastly, the initial ADME profile of some selected derivatives were encouraging. The *in vivo* pharmacokinetic profiles of the most promising compounds in mice were highly encouraging for further optimization of the compound class.

#### Biphenyl Carboxylic Acid

**Figure 16:**
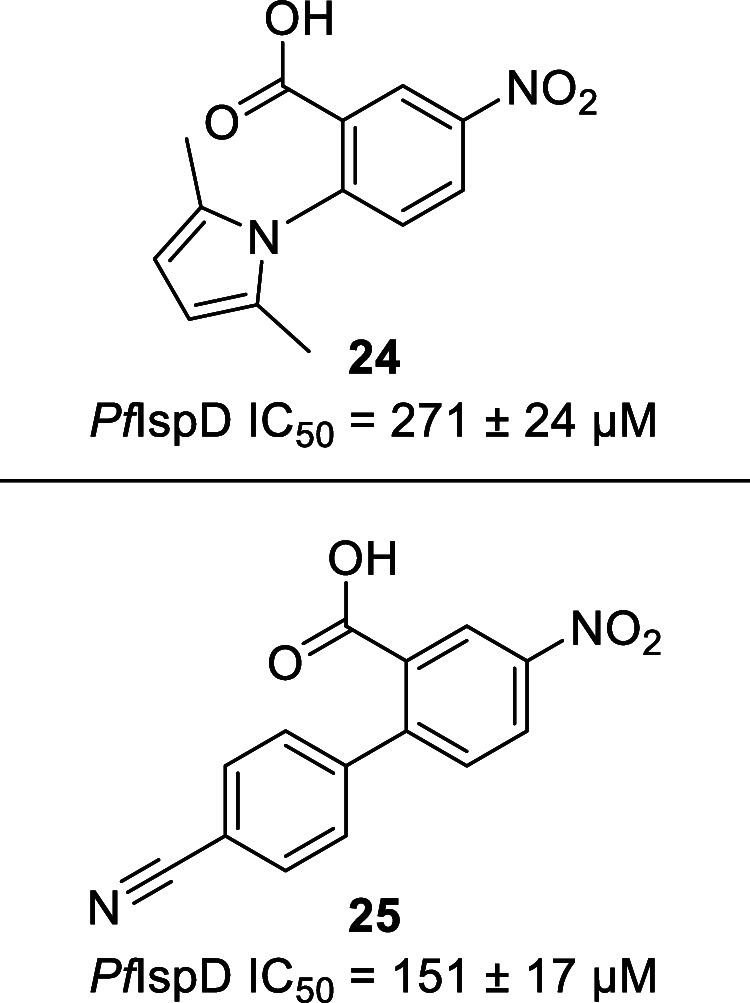
Structures of the initial hit and optimized compound of the biphenyl carboxylic acid, *Plasmodium falciparum* (*Pf*).^42^

The next inhibitor was discovered during an HTS campaign using a proprietary library from BASF.^42^ The authors selected compound **24** because of its fragment-like size that allows plenty of chemical modifications to be made (Figure 16). Besides enhancing the potency and physicochemical properties, the SAR of this fragment also focused on finding a replacement for the pyrrole as this motif might be problematic in a drug-development program. The SAR resulted in compound **25**, which features a good balance between improved potency (*Pf*IspD IC_50_ = 151 ± 17 μM) and physicochemical properties. Furthermore, we were able to replace the pyrrole by a benzonitrile moiety, hence obtaining a more druglike fragment (Figure 16).

#### Aminobenzothiazoles

**Figure 17:**
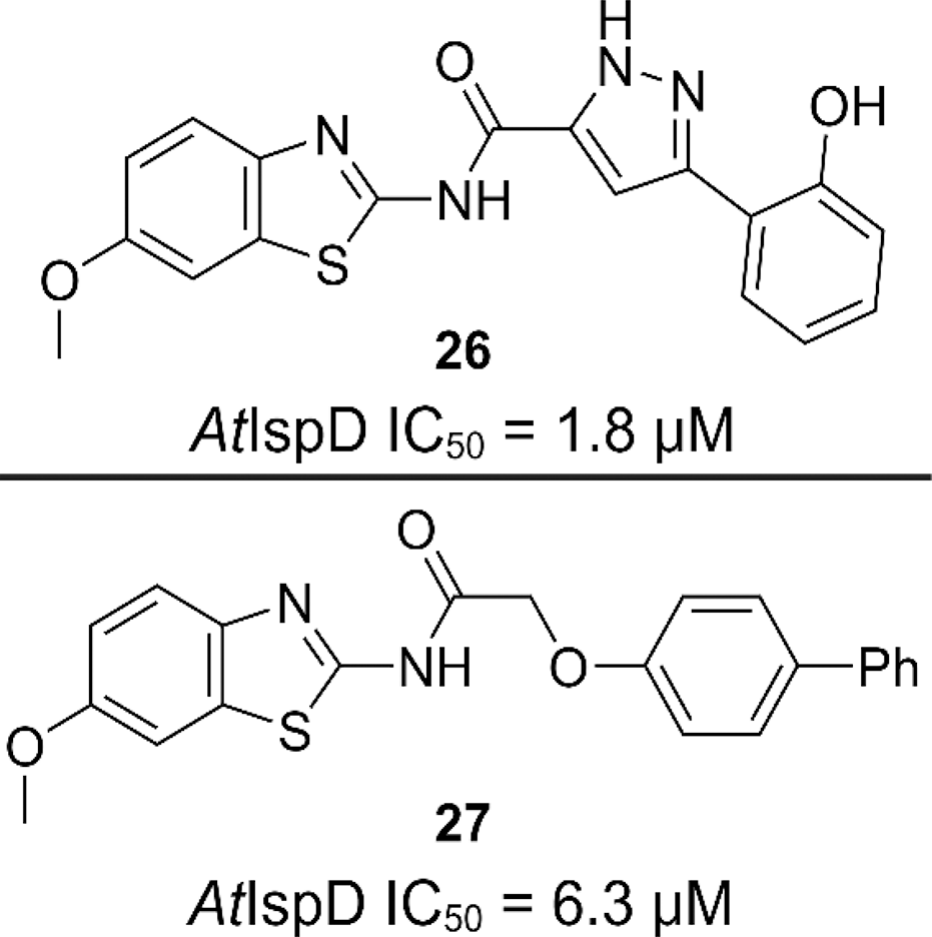
Structures of the initial hits of the aminobenzothiazole inhibitors, *Arabidopsis thaliana* (*At*).^76^

The aminobenzothiazoles (**26** and **27**) were also discovered during screenings at BASF aiming to find *At*IspD inhibitors (Figure 17).^76^ This compound class has low micromolar activity against *At*IspD, although no further optimization of this class was done to this date.

## MEP Mimics

Besides small molecules as IspD inhibitors, various research groups have experimented with close mimics of MEP to influence the enzymatic activity. The aim of these derivatives is to achieve a potentially double working mechanism. On one hand, the analogues could compete with MEP; on the other hand, they could lead to the synthesis of MEP pathway metabolites that are unable to undergo any further conversion to isoprenoids. This approach not only leads to accumulation of purposeless metabolites/products but could also induce metabolic stress as a consequence of the energetic cost of the MEP pathway.

### Desmethylated MEP

**Figure 18:**
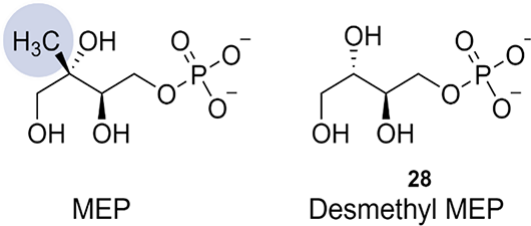
2-*C*-Methyl-d-erythritol 4-phosphate (MEP) and desmethyl MEP.^59^

The most straightforward MEP mimic discovered today is a desmethyl MEP (compound **28** in Figure 18). As the name of the compound suggests, this derivative of MEP lacks the methyl group. Unfortunately, this analogue has a very low potency for *Ec*IspD, displaying an IC_50_ value of only 1.36 mM. Intriguingly, turnover of the compound, albeit with slower kinetics than the natural substrate, toward demethylated CDP-ME was observed.^59^

### Fluoroalkyl Phosphonyl Analogues

**Figure 19:**
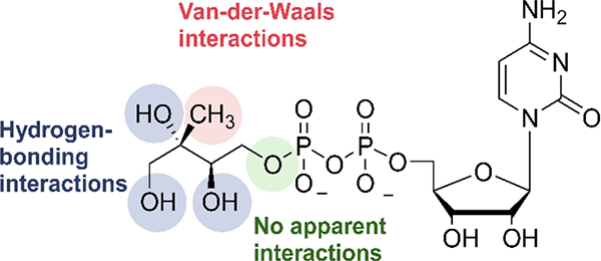
Overview of the interactions of 4-diphosphocytidyl-2-*C*-methyl-d-erythritol (CDP-ME).

**Figure 20:**
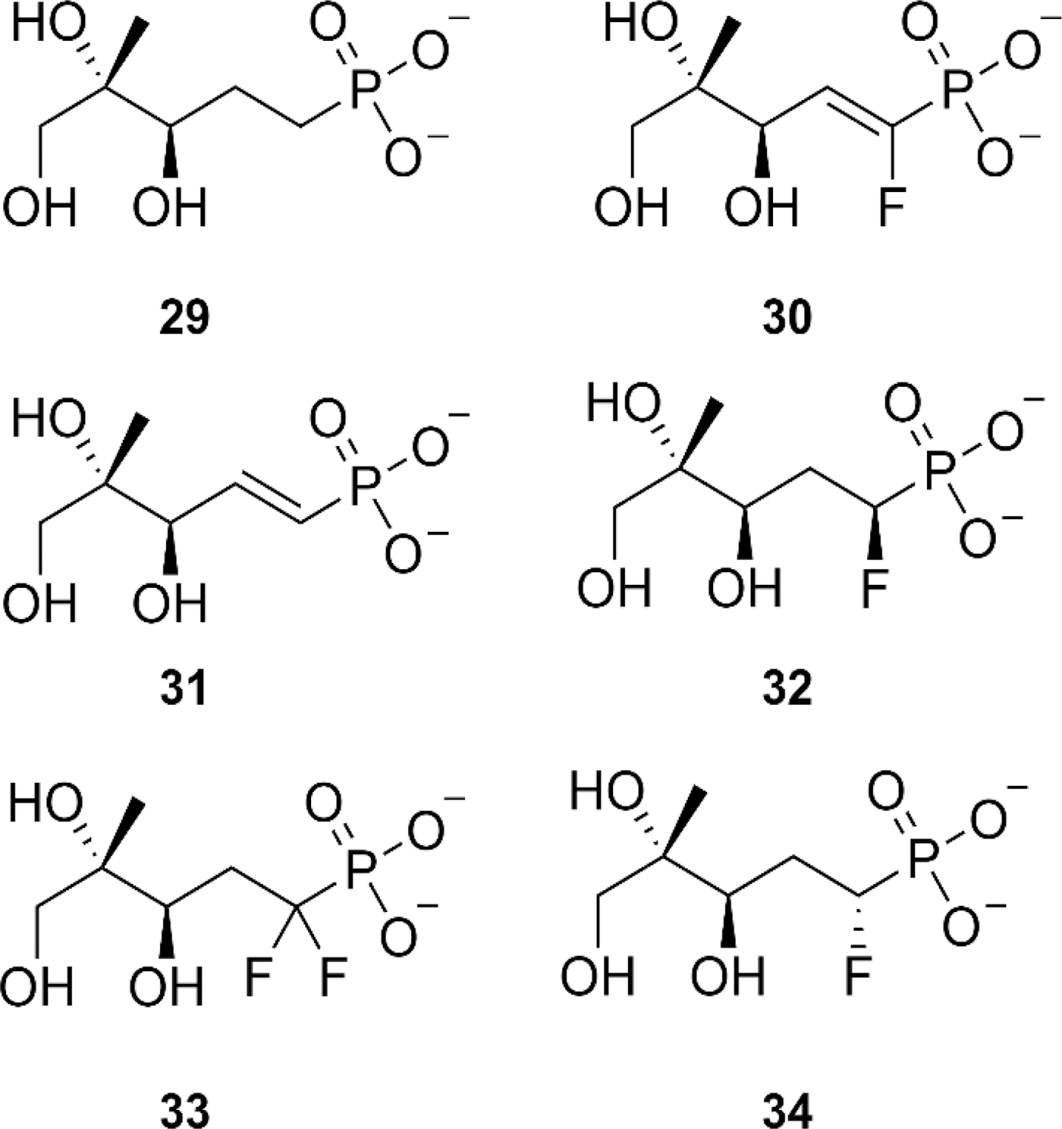
Overview fluoroalkyl phosphonyl analogues of 2-*C*-methyl-d-erythritol 4-phosphate (MEP).^33,60^

Based on previously reported IspD cocrystal structures with CDP-ME (PDB accession code: 1I52), Bartee *et al*. identified the oxygen atom linking the methylerythritol part and the phosphonate as the ideal position for modification. They reasoned that the oxygen is involved in any obvious interactions with the protein interface (Figure 19).^33,60^ Modifications directed at the hydroxyl moieties would cause a loss of hydrogen-bonding interactions, while replacement of the methyl group would lead to a loss of van der Waals interactions (Figure 19). In total, six derivatives in which the oxygen atom was replaced with a carbon atom were synthesized (Figure 20). The ability of these derivatives to serve as substrate was confirmed by LC-MS detection of the corresponding CDP-ME analogues. Despite their ability as substrates, the analogues have significantly lower catalytic efficiencies (*k*_cat_/*K*_m_) in comparison with MEP. The authors postulated that the major factor influencing the catalytic efficiency was the change in reactivity resulting from swapping the oxygen with a carbon atom, resulting in a decreased of turnover (*k*_cat_), and that there was only a minor loss in affinity of the modified substrates toward the protein (*K*_m_). The influence of the analogues on MEP turnover was determined for *Ec*, *Pf*, and *Mt*IspD. It became clear that the derivatives containing saturated linkers performed the best within these experiments, with compound **34** outperforming the rest. IC_50_ values for **34** were determined against *Ec* and *Pf*IspD, being 0.7 ± 0.1 and 1.3 ± 0.7 mM, respectively.

## Conclusions

Despite all efforts made so far, there is only a limited number of IspD inhibitors described in the literature. Furthermore, the existing inhibitors only target a handful of IspD homologues, while IspD is found widespread among human pathogens and plants. As described above, IspD can be inhibited with a wide array of MOIs ranging from competitive over covalent to allosteric inhibition. Taking this into account, there is still a lot of untapped potential.

Future research toward IspD should primarily be focused on obtaining crystallization conditions and identifying the allosteric site of IspD of other species. With these conditions in hand, cocrystal structures with inhibitors should be obtained. This will allow efficient further development toward lead compounds.

We believe that the majority of untapped opportunities for IspD inhibitors are located in the development of anti-infectives. Many of the pathogens flagged as problematic by the recent WHO report covering the bacterial priority pathogens are dependent on the MEP pathway.^77^ This opens a venue to address these pathogens by targeting IspD. Furthermore, due to the overwhelming structural similarities between the different IspD homologues of various pathogens, broad-spectrum anti-infectives targeting IspD could potentially be developed.

In summary, due to its widespread occurrence and absence in human cells, IspD has the potential to play a key role in the quest for novel anti-infectives and herbicides with a unique mode of action.

## ABBREVIATIONS

ADME: absorption, distribution, metabolism, and excretion
AMR: antimicrobial resistance
*At*: *Arabidopsis thaliana*
*Bs*: *Bacillus subtilis*
DXP: 1-deoxyxylulose 5-phosphate
DMADP: dimethylallyl diphosphate
CDP-ME: 4-diphosphocytidyl-2-*C*-methyl-d-erythritol
IspD: 4-diphosphocytidyl-2-*C*-methyl-d-erythritol synthase
*Ec*: *Escherichia coli*
GAP: glyceraldehyde-3-phosphate
HTS: high-throughput screen
Hs: *Homo sapiens*
IDP: isopentenyl diphosphate
MEP: 2-*C*-methylerythritol-d-erythritol-4-phosphate
MVA: mevalonate pathway
*K*_m_: Michaelis constant
MIC: minimum inhibitory concentration
MOI: mode of inhibition
MOA: mode of action
*Ms*: *Mycobacterium smegmatis*
*Mt*: *Mycobacterium tuberculosis*
BITZ: 2-phenyl benzo[d]isothiazol-3(2*H*)-one
*Pf*: *Plasmodium falciparum*
*Pv*: *Plasmodium vivax*
*Pa*: *Pseudomonas aeruginosa*
